# Echinacoside Induces UCP1- and ATP-Dependent Thermogenesis in Beige Adipocytes *via* the Activation of Dopaminergic Receptors

**DOI:** 10.4014/jmb.2306.06041

**Published:** 2023-07-17

**Authors:** Kiros Haddish, Jong Won Yun

**Affiliations:** Department of Biotechnology, Daegu University, Gyeongsan, Gyeongbuk 38453, Republic of Korea

**Keywords:** 3T3-L1, dopaminergic receptors, echinacoside, obesity, thermogenesis

## Abstract

Echinacoside (ECH) is a naturally occurring phenylethanoid glycoside, isolated from *Echinacea angustifolia*, and this study aimed to analyze its effect on thermogenesis and its interaction with dopaminergic receptors 1 and 5 (DRD1 and DRD5) in 3T3-L1 white adipocytes and mice models. We employed RT-PCR, immunoblot, immunofluorescence, a staining method, and an assay kit to determine its impact. ECH showed a substantial increase in browning signals in vitro and a decrease in adipogenic signals in vivo. Additionally, analysis of the iWAT showed that the key genes involved in beiging, mitochondrial biogenesis, and ATP-dependent thermogenesis were upregulated while adipogenesis and lipogenesis genes were downregulated. OXPHOS complexes, Ca^2+^ signaling proteins as well as intracellular Ca^2+^ levels were also upregulated in 3T3-L1 adipocytes following ECH treatment. This was collectively explained by mechanistic studies which showed that ECH mediated the beiging process *via* the DRD1/5-cAMP-PKA and subsequent downstream molecules, whereas it co-mediated the α1-AR-signaling thermogenesis via the DRD1/5/SERCA2b/RyR2/CKmt pathway in 3T3-L1 adipocytes. Animal experiments revealed that there was a 12.28% reduction in body weight gain after the ECH treatment for six weeks. The effects of ECH treatment on adipose tissue can offer more insights into the treatment of obesity and metabolic syndrome.

## Introduction

Obesity is characterized by an imbalance in energy intake and expenditure. Recent advances in molecular biotechnology have brought fresh insights into the involvement of brown and beige fat in obesity and the elucidation of ways to prevent obesity and consequent metabolic disorders [1]. In this regard, the identification of beige subtypes led to the understanding of the additional potential of the brown adipose tissue (BAT) in dissipating energy as heat by the combustion of nutrients during ATP generation via uncoupling protein 1 (UCP1) [2].

UCP1, a unique thermogenic indicator, is one of the 53 members of the mitochondrial carrier family [3] that short-circuits the mitochondrial proton gradient to produce heat via the oxidative phosphorylation of metabolic fuels such as fatty acids [4]. Several lines of evidence suggest that brown and beige adipocytes, despite possessing UCP1, also induce energy expenditure via ATP-consuming Ca^2+^ futile cycles [5, 6]. Catecholamines, under appropriate stimuli, can be engaged to increase intracellular Ca^2+^ cycling by stimulating the ryanodine receptors (RyR) and promoting Ca^2+^ export from the endoplasmic reticulum (ER). Calcium export then raises the activity of the ER Ca^2+^ (sarco/endoplasmic reticulum Ca²+-ATPase2, SERCA2), allowing Ca^2+^ to be imported back into the ER [7]. This cyclic import and export of Ca^2+^ into and from the ER results in the consumption of ATP, which eventually causes energy dissipation that helps to reduce the occurrence of weight gain and protects against obesity.

Many of the synthetic drugs that are still in use today are based on naturally occurring chemicals derived from plants. In the past three decades, around 35% of drugs approved in the global market have been made either directly or indirectly from natural substances [8]. A growing body of evidence suggests that phytochemicals mostly in the group of terpenoids, polyphenols, and alkaloids are excellent alternatives to synthetic drugs that promote adipose tissue browning and may be promising candidates for obesity management [9]. These studies exemplify the role of bioactive compounds in inducing white adipose browning in cultured adipocytes in vitro and in animal models, and this browning effect is linked to various metabolic mechanisms that may help prevent obesity [10, 11].

ECH is a naturally occurring phenylethanoid glycoside with several therapeutic properties, isolated from the perennial herb *Echinacea angustifolia*. ECH has been shown to have beneficial effects in neurological disorders including Parkinson’s and Alzheimer’s diseases [12-14]. It has also been found to contribute to diabetic cardiomyopathy [15] and depressive disorders [16], suppress the hepatitis B virus X protein which plays a role in HBV pathogenesis [17] as well as enhance sperm quantity [18]. ECH has been shown to improve cardiac function [19], display antioxidant properties [20], and suppress cancer [21]. Studies have also reported its role in the inhibition of cardiomyocyte apoptosis [22] and osteoclastogenesis [23], as an antioxidant in aging [24], and in enhancing the immune system [25].

Despite its diverse metabolic and therapeutic functions, the functional properties of ECH in fat browning and ATP-dependent futile cycle processes as well as its interaction with the dopaminergic receptors, have not yet been explored. The aim of this study was, therefore, to examine the functional significance of ECH in UCP1- and ATP-dependent thermogenesis in adipocytes in vitro and in mice in vivo, as well as its interaction with D1-like dopaminergic receptors.

## Materials and Methods 

### Chemicals

ECH and prazosin (an antagonist of α1-adrenergic receptors [AR]) were acquired from Sigma-Aldrich (USA). The D1-like dopaminergic receptor agonist SKF38393 was obtained from Abcam (UK). SCH 23390 (DRD1/5 antagonist) and cirazoline (an α1-AR agonist) were purchased from Tocris Bioscience (UK). All other chemicals employed in this research were of analytical grade.

### MTT Assay

The MTT colorimetric assay, in accordance with the manufacturer's instructions, was employed to determine the percentage of apoptotic cells. Briefly, 3T3-L1 cells were cultured in 24-well plates containing 500 μl Dulbecco’s Modified Eagle’s Medium (DMEM). Following the treatment with different concentrations of ECH, the DMEM medium in each well was substituted with a 200 μl solution containing 0.5 mg/ml MTT. The cells were then incubated at 37°C for 4 h. Afterward, the media were discarded, and the formazan blue generated within the cells was dissolved in 1 ml of DMSO. Optical density was then measured at 540 nm.

### Cell Culture and Differentiation

3T3-L1 preadipocytes obtained from ATCC (USA) were cultured in DMEM (Thermo Fisher Scientific Inc., USA) supplemented with 10% fetal bovine serum (FBS; Thermo Fisher Scientific Inc.) and 100 μg/ml penicillin-streptomycin (Invitrogen, USA). The cells were cultured for 7-10 passages at 37°C in a 5% CO_2_ incubator. A suitable quantity of confluent 3T3-L1 cells was cultured in the differentiation induction medium composed of 10 μg/ml insulin (Sigma-Aldrich), 0.25 mM dexamethasone (Sigma-Aldrich), and 0.5 mM 3-isobutyl-1-methylxanthine (Sigma-Aldrich) in DMEM for 48 h. Subsequently, the cells were cultured for an additional 72 h in a DMEM maturation medium supplemented with 10% FBS and 10 μg/ml insulin.

### Animal Experiments

For all the experiments, five-week-old C57BL/6 mice were purchased from Hyochang Science (Korea) and housed individually. The mice were maintained under a 12-h light/dark cycle at a temperature of 27°C with 34%humidity. They were acclimatized to a regular chow diet for 1 month, followed by a high-fat diet (HFD, 60% fat) for 5 weeks. Afterward, the mice were divided into two groups: HFD-fed control mice (HFD, *n* = 6) and HFD-fed mice treated with ECH (HFD + ECH, *n* = 6). Food was provided every three days. Both groups of mice were maintained on a high-fat diet (HFD), and in the HFD + ECH group while ECH was administered intraperitoneally every three days at a dosage of 25 mg/kg body weight for a duration of 6 weeks. Body weight measurements were recorded every three days throughout the experiment. All animal experiments were approved by the Committee for Laboratory Animal Care and Use of Daegu University (DUIACC-2022-15-0901-015).

### Hematoxylin and Eosin Staining

A histological examination was conducted on the iWAT, eWAT, and BAT. The tissues were fixed in 10% neutral-buffered formalin, followed by washing with phosphate-buffered saline (PBS), and then embedded in paraffin wax. Sections of the paraffin-embedded tissues, approximately 3-5 μm thick each, were deparaffinized, rehydrated, and stained with hematoxylin (Vector Laboratories Inc., Canada) and eosin (H&E, Daejung, Korea). Optical microscopy using an Olympus IX51 microscope (Japan) was employed to conduct the histopathological analysis of the tissues.

### Quantitative Real-Time RT-PCR

Total RNA was isolated from the iWAT using a total RNA isolation kit (RNA-spin, iNtRON Biotechnology, Korea). Subsequently, cDNA synthesis was performed using 1 μg of RNA and a Maxime RT premix (iNtRON Biotechnology). The mRNA levels were quantitatively assessed using qPCR (Stratagene 246 mx 3000p QPCR System, Agilent Technologies, USA) with SYBR Green (Roche, Switzerland) staining. All experiments were conducted with technical triplicates, and the mRNA expression levels were normalized to the β-actin gene levels in the corresponding RNA samples. The primer sequences used in this study are provided in Table 1.

### Western Blot Analysis

Cell lysates were prepared by adding a radioimmunoprecipitation assay buffer (RIPA buffer, Sigma–Aldrich), followed by homogenization and centrifugation at 13,000 ×*g* for 30 min. The cell extract was diluted in 5× sample buffer, which consisted of 50 mM Tris at pH 6.8, 2% sodium dodecyl sulfate (SDS), 10% glycerol, 5% β-mercaptoethanol, and 0.1% bromophenol blue. The mixture was then heated at 95°C for 5 minutes. Subsequently, the samples were subjected to 8%, 10%, or 12% sodium dodecyl sulfate-polyacrylamide gel electrophoresis (SDS-PAGE). After electrophoresis, the proteins were transferred to a polyvinylidene difluoride membrane (PVDF, Santa Cruz Biotechnology, USA). The membrane was then blocked for 1 h using TBS-T (10 mM Tris-HCl, 150 mM NaCl, and 0.1% Tween 20) containing either 5% skim milk or bovine serum albumin (BSA) (Rocky Mountain Biologicals, USA). Membranes were cut horizontally for proteins with a wide range of molecular weights relative to β-actin, while proteins with extremely close molecular weights were cut vertically. The membrane was rinsed three times consecutively with TBS-T buffer, followed by 1 h incubation with a 1:1000 dilution of various primary polyclonal antibodies: [anti-β-actin, anti-DRD1, anti-DRD5, anti-ATGL, anti-ACC, anti-ATF2, anti-ACOX1, anti-C/EBPα, anti-FAS, anti-p-ACC, anti-PKA, anti-p38, anti-p-p38, anti-PPARγ, anti-AMPK, anti-ATF2, anti-p-ATF2, anti-CREB, anti-VDAC, anti-p-CREB, anti-ERK1/2, anti-p-ERK1/2, anti-PGC-1α, anti-UCP1, anti-PPARα, anti-ATP5B (Santa Cruz Biotechnology)], [anti-p-AMPK, anti-α1-AR (Invitrogen)],[anti-MCU, anti-pHSL, anti-SERCA2b (Cell Signaling Technology)], [anti-PRDM16, anti-CPT1, anti-CYT-C, anti-CaMKII, anti-PDE4, anti-OXPHOS (Abcam], [anti-RyR2 and anti-CKmt (Proteintech)]. After washing the probed membranes twice, they were incubated for 1 hour with a horseradish peroxidase-conjugated anti-rabbit IgG or anti-mouse IgG secondary antibody in TBS-T buffer supplemented with 1% BSA. The membranes were then detected using enhanced chemiluminescence with an ImageQuant LAS500 cooled CCD imager (GE, USA). The intensity of the protein bands was quantified using the ImageJ software (NIH).

### Immunocytochemical Analysis

To directly detect the expression of UCP1 in the control and ECH-treated adipocytes, immunocytochemical analysis was performed on formalin-fixed 3T3-L1 cells. The obtained sections were incubated overnight at 4°C with the primary UCP1 antibody (dilution 1:1000, Santa Cruz Biotechnology), followed by incubation with the appropriate fluorescein isothiocyanate (FITC) goat anti-mouse secondary antibody at room temperature for 4 h. The mitochondria were stained by adding MitoTracker red (1 mM, Cell Signaling Technology, USA) to PBB-T (PBS+1% BSA, and 0.1% Tween 20) at a concentration of 200 nM, and incubating the cells for 2 h at 37°C. Following incubation, the cells were washed with PBS and subjected to immunostaining. The morphological findings were observed using an optical microscope at ×40 magnification.

### Detection of Intracellular Ca^2+^

Calcium Quantification Kit-Red Fluorescence (Abcam, cat#: ab112115) was employed to measure intracellular calcium. Briefly, the calcium standard was first prepared following the manufacturer’s protocol by diluting the appropriate amount of the 300 mM calcium standard in deionized water to produce a calcium concentration ranging from 0 to 3 mM (12 mg/dl). Next, 50 μl of serially diluted calcium standard was added into each well. Then, 50 μl of the assay reaction mixture was added to each well of the calcium standard, blank control, and test samples to make up the total calcium assay volume of 100 μl/well. Finally, the reaction was incubated for 20–30 min at room temperature, protected from light. Fluorescence intensity was monitored with a fluorescence plate reader at excitation/emission (Ex/Em) wavelengths of 540/590 nm.

### Statistical Analysis

All the data are presented as the mean ± standard deviation (SD). At least three independent experiments were performed for each data set. The Statistical Package of Social Science (SPSS, version 17.0; SPSS Inc., USA) tool was used to examine the statistical significance among several groups. The Student’s *t*-test was used to assess the data for only two groups. A Tukey’s post-hoc test evaluated the differences between more than three experimental groups using one-way or two-way ANOVA; *p* values < 0.05 or 0.01 were considered significant.

## Results

### Echinacoside Reduces Diet-Induced Obesity in Mice

**Fig. 1:**
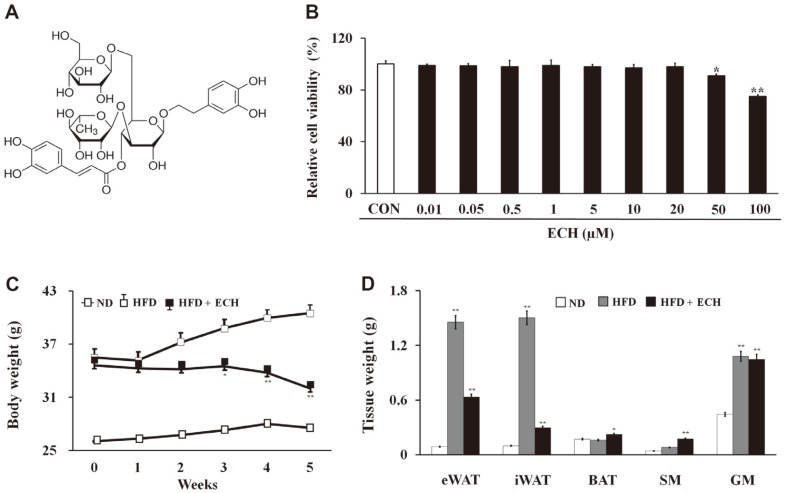
Echinacoside reduces diet-induced obesity in mice. Structure of ECH (**A**). Cytotoxicity of ECH on 3T3-L1 cells as determined by an MTT assay (**B**). Body weight of mice observed over 6 weeks (**C**). Relative weight of various tissues in HFDfed mice and ECH-treated HFD-fed mice (**D**). Histograms represent the results of triple-independent experiments for immunoblot analysis and are presented as the mean ± SD. Differences between groups were determined using the Statistical Package for the Social Sciences (SPSS; version 17.0; SPSS Inc., USA) to perform ANOVA followed by Tukey's post-hoc tests. Statistical significance between control and ECH-treated 3T3-L1 cells/tissue is shown by *p < 0.05 or **p < 0.01.

We first applied an MTT assay to assess the cytotoxicity of ECH (Fig. 1A) on 3T3-L1 cells and found no cytotoxicity up to 20 μM. A final concentration of 10 μM was selected as the working amount for subsequent in vitro experiments (Fig. 1B). Next, we investigated the effect of ECH on body weight and adipose tissue in vivo. 25 mg/kg of ECH was intraperitoneally injected into the HFD-fed mice, twice a week, for 6 weeks and a substantial decline in relative body weight was observed starting from the second week (Fig. 1C). We also measured the weights of various tissues. There was a significant reduction in eWAT and iWAT mass in HFD-fed mice after the ECH treatment, whereas a significant increase in BAT and skeletal muscle mass was observed as indicated in Fig. 1D.

### Echinacoside Induces Beiging in 3T3-L1 Adipocytes

**Fig. 2:**
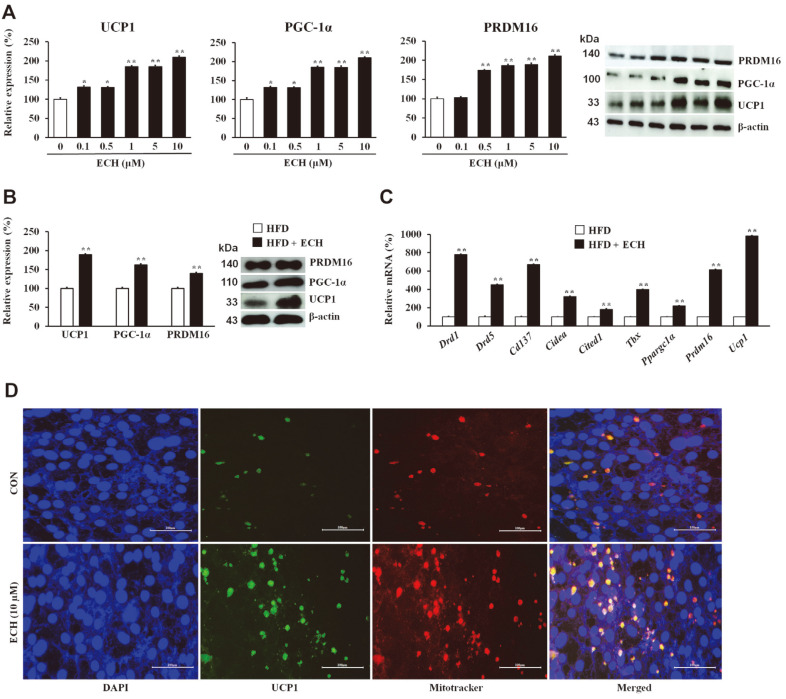
Echinacoside induces beiging in 3T3-L1 adipocytes. Effect of ECH on key browning proteins (A and B). mRNA expressions of the core set of beige-fat marker genes in ECH-treated HFD-fed mice (**C**). Immunohistochemical staining in 3T3-L1 cells (**D**) (×40 magnification; scale bar = 100 μm). Histograms represent the results of triple-independent experiments for immunoblot analysis and are presented as the mean ± SD. Differences between groups were determined using the Statistical Package for the Social Sciences (SPSS; version 17.0; SPSS Inc., USA) to perform ANOVA followed by Tukey's post-hoc tests. Statistical significance between control and ECH-treated 3T3-L1 cells/tissue is shown by *p < 0.05 or **p < 0.01.

Subsequently, we investigated the impact of ECH on key marker proteins associated with browning (UCP1, PGC-1α, and PRDM16). Our findings demonstrated that ECH treatment resulted in a significant and dose-dependent increase in the expression of these core browning marker proteins (Fig. 2A and 2B). In addition, the administration of ECH to HFD-fed mice displayed rapid upregulation of BAT-specific genes (*Cd137*, *Cidea*, *Cited1*, *Tbx*, *Ppargc1a*, *Prdm16*, and *Ucp1*) in iWAT (Fig. 2C). Furthermore, the staining of differentiated 3T3-L1 white adipocytes with MitoTracker Red showed stronger signals in the ECH-treated adipocytes than in the control cells, validating the positive regulation of ECH in the browning process (Fig. 2D).

### Echinacoside Promotes Lipid Metabolisms and Fat Oxidation

**Fig. 3:**
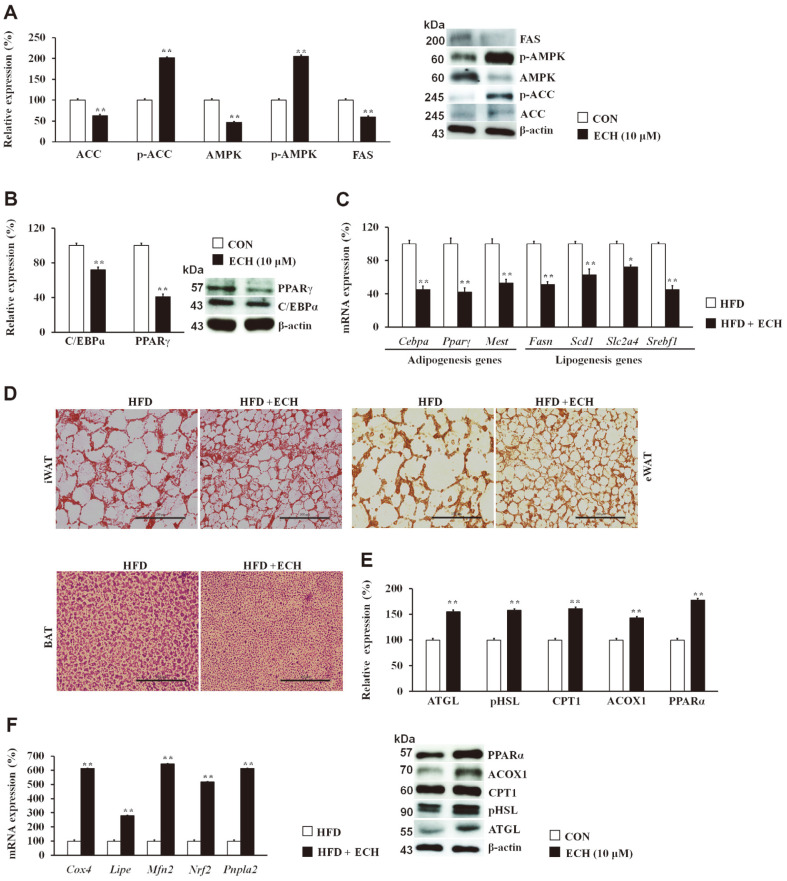
Echinacoside promotes lipid metabolism in 3T3-L1 white adipocytes. Effect of ECH in the expression levels of lipogenesis (**A**) and adipogenesis markers (**B**). Effect of ECH on the mRNA expression of key adipogenesis and lipogenesis genes in ECH-treated HFD-fed mice (**C**). H & E staining for inguinal white adipose tissue (iWAT), epididymal adipose tissue (eWAT), and brown adipose tissue (BAT) (**D**). Effect of ECH in lipolysis and fat oxidation markers (**E**) in 3T3-L1 cells. mRNA expression of key lipolysis and fatty acid oxidation genes in ECH-treated HFD-fed mice (**F**). Histograms represent the results of triple-independent experiments for immunoblot analysis and are presented as the mean ± SD. Differences between groups were determined using Student’s *t*-test. Statistical significance between control and ECH-treated 3T3-L1 cells/tissue is shown by *p < 0.05 or **p < 0.01.

Building upon its application, we examined whether ECH affects lipid metabolism in 3T3-L1 white adipocytes. To investigate this, we first determined the expression level of acetyl-CoA carboxylase, which was downregulated after ECH treatment, with a concurrent increase in phosphorylated (p-ACC) and p-AMPK. Fatty acid synthase (FAS) was also downregulated after the ECH treatment of 3T3-L1 white adipocytes (Fig. 3A). In addition, adipogenic transcriptional factors (C/EBP and PPARγ) were downregulated following the ECH treatment of the 3T3-L1 cells (Fig. 3B). The mRNA expression levels of key genes associated with adipogenesis and lipogenesis were also downregulated following the ECH treatment in HFD-fed mice (Fig. 3C). This was further elucidated by the histopathological analysis determined by H & E staining in iWAT, eWAT, and BAT, which showed a reduction in lipid cell size in the ECH + HFD treated group compared with the HFD counterparts as indicated in Fig. 3D. The expression levels of lipolysis and fat oxidation-related proteins were also examined. ECH increased lipolysis by raising the levels of phosphorylated hormone-sensitive lipase (p-HSL) and adipose triglyceride lipase (ATGL). It also resulted in a notable increase in the expression of acyl-coenzyme A oxidase 1 (ACOX1), carnitine palmitoyltransferase 1 (CPT1), and PPARα, suggesting an enhanced ability of ECH to facilitate fat oxidation (Fig. 3E). Moreover, core genes for the lipolysis and fat oxidation processes were also substantially upregulated with the ECH treatment of the HFD-fed mice (Fig. 3F).

### Echinacoside Upregulates Calcium Cycling Proteins, Increases Intracellular Calcium, and Facilitates Mitochondrial Biogenesis

**Fig. 4:**
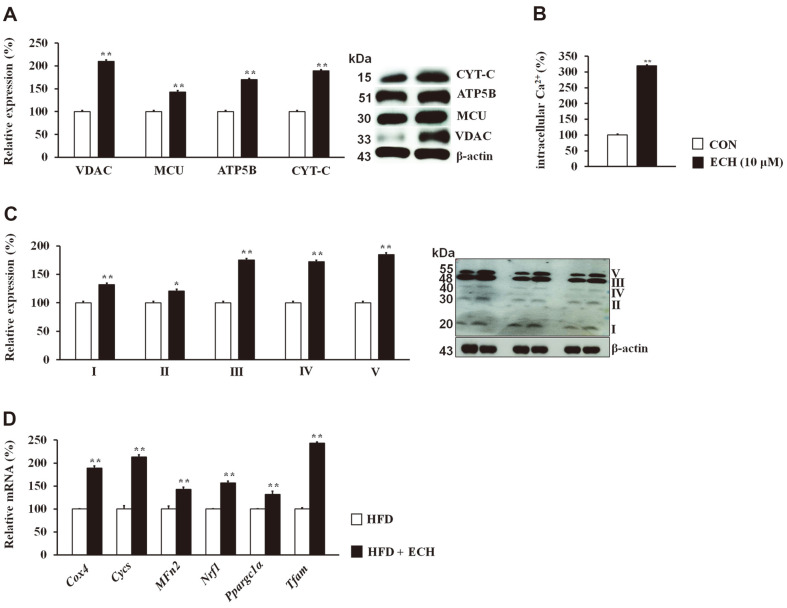
Echinacoside facilitates mitochondrial biogenesis. Effect of ECH on the mitochondrial and ATP synthesis effectors (**A**). Effect of ECH on intracellular calcium (**B**). Effect of ECH on mitochondrial oxidative phosphorylation (OXPHOS) complexes (I, II, III, IV, and V) (**C**). In vivo effect of ECH on mitochondrial biogenesis genes (**D**). Histograms display triple independent experiments for immunoblot analysis and are presented as the mean ± SD. Differences between groups were determined using Student's *t*-test. Statistical significance between control and ECH-treated 3T3-L1 cells/tissue is shown by *p < 0.05 or **p < 0.01.

We next sought to understand the role of ECH in the regulation of calcium-cycling proteins and intracellular calcium levels as well as mitochondrial biogenesis and found that ECH upregulated voltage-dependent anion (VDAC), mitochondrial calcium uniporter (MCU), ATP synthase F1 subunit beta (ATP5B), and cytochrome C (CYT-C) in 3T3-L1 cells (Fig. 4A). Intracellular calcium was also found to be significantly increased following the ECH treatment of 3T3-L1 cells (Fig. 4B). As shown in Fig. 4C, OXPHOS complexes (I, II, III, IV, and V) were upregulated in the ECH-treated 3T3-L1 cells. This was collectively elucidated in vivo by measuring the mRNA expression levels of mitochondrial biogenesis genes including *Cox4*, *Cycs*, *Nrf1*, *Mfn2*, *Tfam*, and *Ppargc1α*, all of which were remarkably increased following the ECH treatment of the HFD-fed mice (Fig. 4D).

### Echinacoside Induces Browning of White Adipocytes via the DRD1/5/PKA/p38 MAPK Pathway

**Fig. 5:**
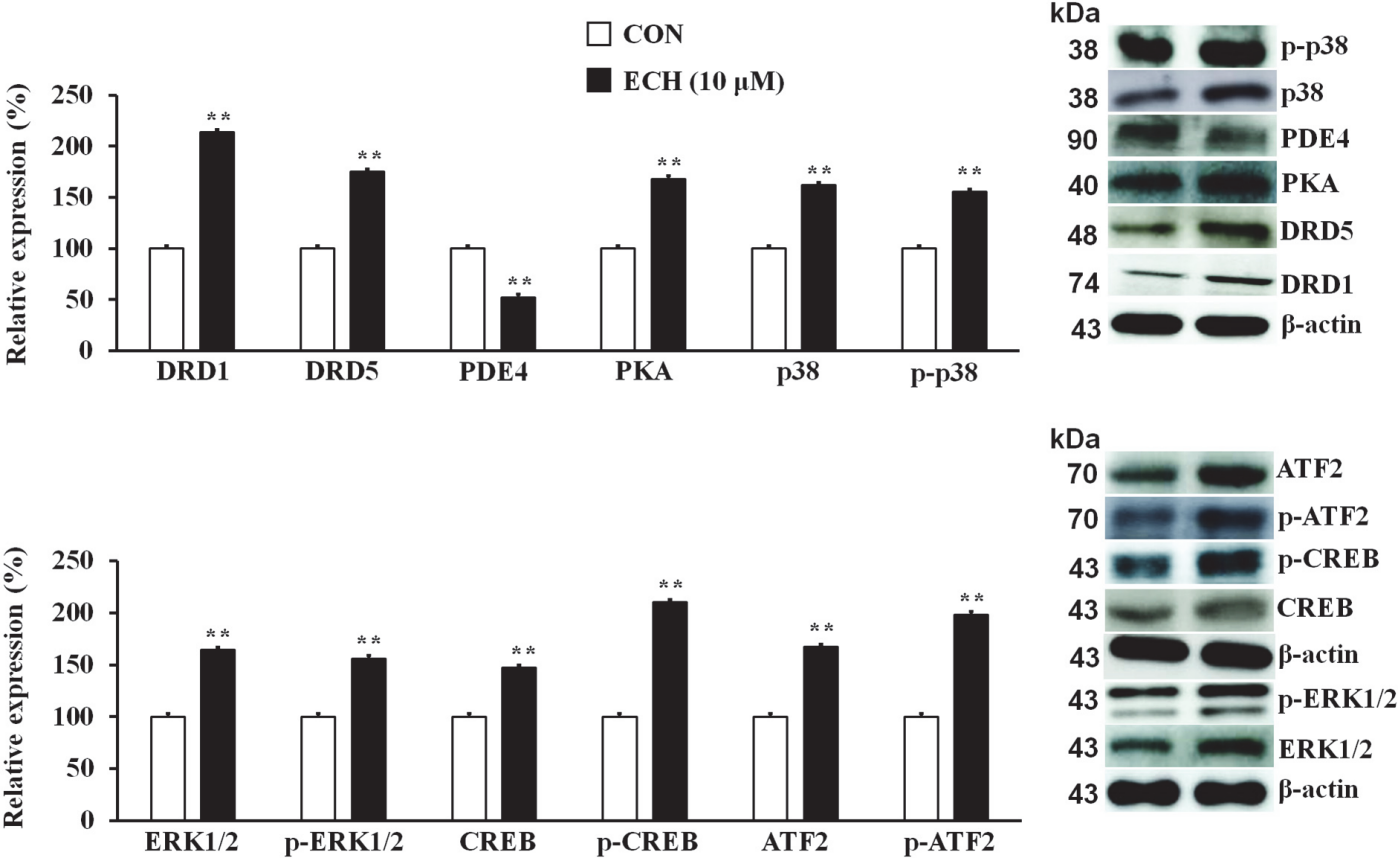
Echinacoside upregulates browning pathway markers in 3T3-L1 adipocytes. Effect of ECH on beiging pathway molecules. The histograms represent the results of triple-independent experiments for immunoblot analysis and are presented as the mean ± SD. The differences between the groups were determined using the Student’s t-test. Statistical significance between control and ECH-treated 3T3-L1 cells is indicated by *p < 0.05 or **p < 0.01. Statistical significance between control and ECH-treated 3T3-L1 cells/tissue is shown by *p < 0.05.

**Fig. 6:**
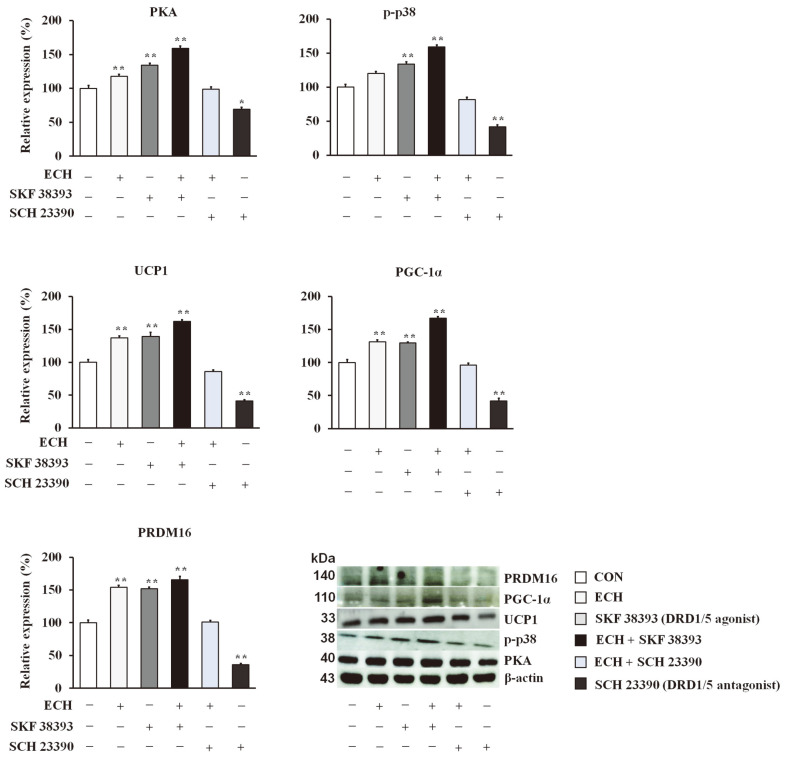
Mechanistic studies for the effect of echinacoside on browning effectors in 3T3-L1 cells. Effect of ECH or SKF38393 (DRD5 agonist) and SCH 23390 (DRD1 antagonist) in pathway molecules. The histograms represent the results of triple-independent experiments for immunoblot analysis and are presented as the mean ± SD. The differences between the groups were determined using the Statistical Package for the Social Sciences (SPSS; version 17.0; SPSS Inc., USA) to perform ANOVA, followed by Tukey’s post-hoc tests. Statistical significance between control and ECH-treated 3T3-L1 cells/tissue is shown by *p < 0.05 or **p < 0.01.

To unravel the underlying mechanisms by which ECH contributes to the beiging of white adipocytes, we conducted further investigations. Following this, ECH treatment substantially increased the expression levels of signaling molecules, such as DRD1, DRD5, protein kinase A (PKA), p38 mitogen-activated protein kinase (p38 MAPK), phosphorylated p38 (p-p38), extracellular signal-regulated kinase (ERK)1/2, phosphorylated (p)-ERK1/ 2, cAMP-response element binding protein (CREB), p-CREB, activating transcription factor 2 (ATF2) and p-ATF2 in 3T3-L1 adipocytes (Fig. 5). A mechanistic study was undertaken to elucidate the participation of these pathway molecules by treating cells with SKF38393 (DRD1/5 agonist) and SCH 23390 (DRD1/5 antagonist) alone or together with ECH. The results showed that SKF38393 stimulated the expression levels of these molecules along with the browning effectors (UCP1, PGC-1α, and PRDM16), whereas SCH 23390 reversed the agonistic action of SKF38393 (Fig. 6).

### Echinacoside Stimulates α1-AR-Mediated ATP-Dependent Thermogenesis by the Activation of DRD1/5

**Fig. 7:**
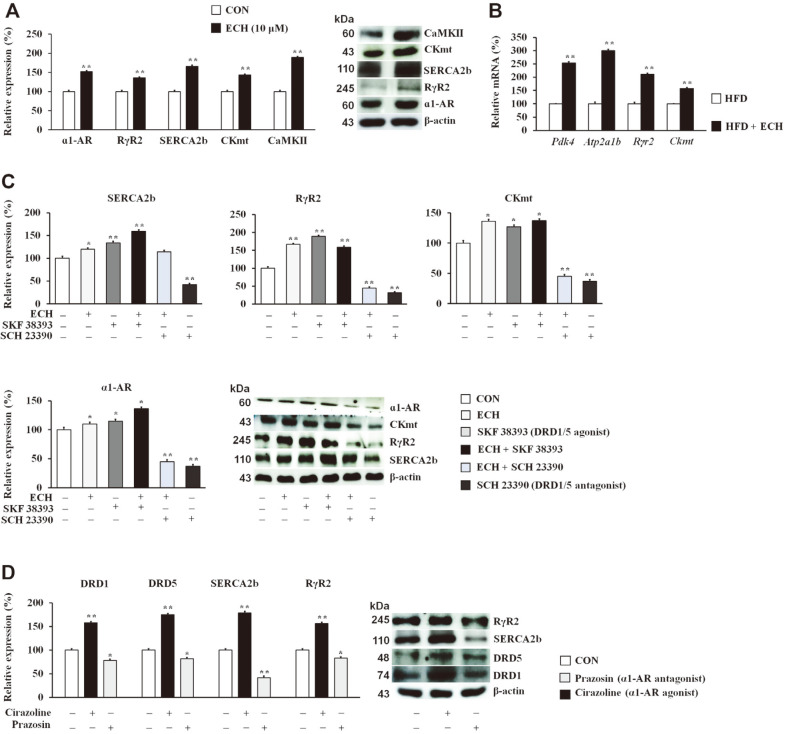
Echinacoside stimulates ATP-dependent thermogenesis. Effect of ECH on ATP-dependent thermogenic mechanism effectors in 3T3-L1 cells (**A**). Effect of ECH on the mRNA expressions of genes involved in UCP1-independent thermogenesis (**B**). Effect of ECH and or SKF38393 (DRD1/5 agonist) as well as SCH 23390 (DRD1/5 antagonist) on ATPdependent thermogenic mechanism effectors (**C**). Effect of prazosin (α1-AR antagonist) cirazoline (α1-AR agonist) on DRD1 and DRD5 as well as ATP-dependent thermogenic mechanisms effectors (**D**). Histograms represent the results of tripleindependent experiments for immunoblot analysis and are presented as the mean ± SD. Differences between groups were determined using the Statistical Package for the Social Sciences (SPSS; version 17.0; SPSS Inc., USA) to perform ANOVA followed by Tukey's post-hoc tests or Student's *t*-test. Statistical significance between control and ECH-treated 3T3-L1 cells/ tissue is shown by *p < 0.05 or **p < 0.01.

Finally, we investigated the impact of ECH on the ATP-dependent thermogenic mechanisms in 3T3-L1 cells. ECH treatment substantially increased the expression levels of the ryanodine receptor (RyR)2, sarco/endoplasmic reticulum Ca^2+^-ATPase (SERCA)2b, mitochondrial creatine kinase (CKmt), and Ca^2+^/calmodulin-dependent protein kinase II (CaMKII) as well as their upstream receptor, α1-AR (Fig. 7A). The mRNA expressions of the genes involved in the ATP-dependent thermogenesis were also dramatically increased in the ECH-treated HFD-fed mice (Fig. 7B). This was further demonstrated by a mechanistic study showing that SKF38393 (DRD1/5 agonist) alone or together with ECH stimulated the expression levels of these proteins, whereas SCH 23390 (DRD1/5 antagonist) reversed the agonistic action of SKF38393 (Fig. 7C). We also sought to investigate the interaction of DRD1 and DRD5 with α1-AR and found that prazosin (α1-AR antagonist) downregulated DRD1, DRD5, and their common downstream effectors while cirazoline (α1-AR agonist) reversed the antagonistic effect of prazosin (Fig. 7D).

## Discussion

A growing body of evidence indicates that phytochemicals mediate thermogenesis via the stimulation of the β3-AR [9, 26, 27]. These molecules target various pathways that are intricately linked to the process of adipogenesis, lipogenesis, lipolysis, and the fat oxidation process, all of which provide a fertile environment for high energy expenditure in the metabolic machinery [11, 28]. As a part of this energy dissipation process, we aimed to investigate alternative receptors that promote thermogenesis and the ATP-dependent futile cycle process when activated with similar natural compounds. In this study, we found that ECH, a naturally occurring phenylethanoid glycoside isolated from *Echinacea angustifolia*, a species of flowering plant of the family Asteraceae, promoted thermogenesis via the activation of D1-like dopaminergic receptors and prevented obesity.

AMP-activated protein kinase (AMPK), the primary energy sensor that monitors the catabolic and anabolic energy balance [29], phosphorylates ACC, a cascade that inhibits fatty acid synthesis and enhances fatty acid oxidation [30]. In this study, the ECH treatment decreased ACC and FAS while increasing p-AMPK and p-ACC, indicating its negative regulation of lipogenesis-related proteins in 3T3-L1 preadipocytes. The current study also revealed that the ECH treatment downregulated the core adipogenic transcription factors (C/EBPα and PPARγ), suggesting its inhibiting role in adipogenesis. The effect of ECH in our current study is consistent with the role of various phytochemicals in the literature that inhibits adipogenesis and lipogenesis [9, 26, 27].

The cyclic AMP/protein kinase A/hormone sensitive lipase (cAMP/PKA/HSL) signaling pathway plays a major role in lipolysis which leads to the formation of free fatty acids (FFA) [31]. This pathway generates FFAs through lipolysis which regulates the energy expenditure of the body and serves as fuel for energy generation in adipocytes [32]. In our study, ECH upregulated the major lipolysis and fat oxidation mediating molecules as well as effectors suggesting its positive role in lipid catabolism.

The cAMP/PKA/p38 MAPK/ERK signaling pathway is the most familiar pathway for the β-oxidation of fatty acids and browning of white adipocytes [33,34]. In this process, catecholamines stimulate adenylyl cyclase (AC), which raises cAMP and subsequently binds to PKA. The PKA-mediated phosphorylation cascade enhances lipolysis, activates p38 MAPK and CREB, and this signaling pathway consequently promotes a thermogenic program. In parallel, the p38 MAPK phosphorylates and activates the transcription factor ATF2 and core browning effectors, causing the transcription of downstream thermogenic genes to be activated [33]. In our study, ECH stimulated the browning of 3T3-L1 adipocytes via the activation of DRD1/5, suggesting that dopaminergic receptors could be an alternative target for anti-obesity drugs in the adipose tissue. This is consistent with our recent findings that L-dihydroxyphenylalanine stimulated the browning of 3T3-L1 adipocytes *via* the activation of the DRD1 and the β3-AR pathways [35], and these receptors were found to synergistically mediate browning in 3T3-L1 cells [36]. To date, numerous studies have illustrated the interaction of phytochemicals with dopaminergic receptors, but their primary focus has been on the interference with diseases of the central nervous system (CNS)[37-39].

One of the major findings of our study was the elucidation of the role of ECH and DRD1/5 in mitochondrial biogenesis and related activities. Mitochondria are the primary source of energy for cellular activity through ATP generation via oxidative phosphorylation, and to operate the various intracellular signaling cascades, mitochondrial biogenesis must occur continuously through self-renewal [40]. The decrease in mitochondrial biogenesis, oxidative metabolic pathways, and OXPHOS complexes in adipose tissue indicate that mitochondrial dysfunction or low mitochondrial number and activity are risk factors in obesity and related metabolic disorders [41]. In this study, ECH upregulated mitochondrial biogenesis genes and proteins as well as OXPHOS complexes *in vivo* and *in vitro* indicating its positive regulation of mitochondrial biogenesis. It has been demonstrated in earlier reports that dopaminergic receptors positively regulate mitochondrial biogenesis and function. For example, in behavioral function studies, the DRD1 agonist upregulated mitochondrial biogenesis in 6-hydroxydopamine lesioned rat models, and the agonistic effects were attenuated by the administration of the DRD1 antagonist [42]. Dopamine upregulated mitochondrial mass and thermogenesis in BAT through the D1-like dopaminergic receptors [43]. SKF38393, a DRD1/5 agonist, promoted mitochondrial transport in cultured hippocampal neurons [44]. Our recent studies also indicated that silencing of DRD4 increased the mitochondrial biogenesis effectors [45], while the knockdown of DRD5 exhibited downregulation of these effectors including the OXPHOS complexes in 3T3-L1 and C2C12 cells [46], suggesting their opposite effect on mitochondrial biogenesis. However, further research is required to investigate the effect of ECH on OXPHOS complexes, specifically complex V (ATP synthase). This investigation should include examining the interaction between ECH and oligomycin (ATP synthase inhibitor), as well as other assays that specifically target the biochemical aspects of mitochondria in order to verify whether the upregulation of complex V occurs independently of ECH.

Another remarkable finding from our study is that ECH positively regulated the creatine-dependent ADP/ATP substrate cycling and α1-AR-mediated ATP-dependent thermogenesis in beige adipocytes. The creatine kinase/phosphocreatine (CK/PCr) shuttle enhances energy transfer between mitochondrial and/or glycolytic ATP supply and consumption at the ER by SERCA2b [47]. In addition, UCP1-independent thermogenesis depends on the ATP-dependent Ca^2+^ cycling fashion in which SERCA2b and RyR2 monitor Ca^2+^ import/export, which eventually causes energy dissipation [5, 48, 49]. Our present study revealed that ECH stimulated DRD1/5 and activated ATP-dependent thermogenesis *via* the DRD1/5/SERCA2b/RyR2 and creatine-dependent ADP/ATP substrate cycling pathways in beige adipocytes. Our study not only identified DRD1 and DRD5 as potential targets for ECH but also demonstrated that these receptors interact with α1-AR to regulate the ATP-consuming futile cycles in beige adipocytes.

Our study also revealed the positive association between ECH, DRD1, and DRD5 as well as calcium regulatory proteins in beige adipocytes. Ca^2+^ signaling is essential in improving the metabolic apparatus by boosting calorie intake, thereby minimizing the manifestation of obesity [50, 51]. The association between calcium signaling proteins (VDAC and MCU) is already established, with VDAC being upstream of MCU, as Ca^2+^ must travel through the outer mitochondrial membrane, where VDAC is located before reaching the MCU [52]. In our study, ECH substantially increased VDAC and MCU as well as the intracellular Ca^2+^ level in 3T3-L1 cells. In line with our study, impaired dopamine homeostasis remarkably lowered the VDAC1 and VDAC2 levels in human neuroblastoma SH-SY5Y cells [41], suggesting the positive modulation of dopaminergic receptors to VDAC even if the specific dopaminergic subtypes responsible to mediate this process were not clarified. Other earlier studies also reported that the pharmacological activation of DRD1 and DRD5 modified the synaptic function of the hippocampal and neocortical neurons *via* Ca^2+^ and cAMP-dependent signaling [53] to control the Ca^2+^ channel activity in neurons originating from the dorsal striatum [54].

Finally, our study revealed that ECH positively regulated ATP5B in beige adipocytes. This could be because of the constant entry of cytosolic Ca^2+^ through the VDAC/MCU gate, and once within the mitochondria, these calcium ions modulate cell survival and ATP production [55]. Taken together, our study indicated that ECH not only directly enhances ATP5B expression, but also led to the expectation that increased levels of VDAC and MCU could positively regulate ATP5B. In accordance with the objective of our study, we made efforts to address the limitations; however, the unavailability of pharmacological drugs that can entirely distinguish between DRD1 and DRD5 prevented us from determining the specific affinity of ECH for either receptor. Nevertheless, this study explores the activity of ECH and the functional roles of dopaminergic receptors in browning activities. This research provides the concept that dopaminergic receptors can serve as alternative receptors for the thermogenesis process, presenting a potential alternative strategy for combating obesity. However, to establish a more comprehensive understanding, further investigations, including detailed *in vivo* research and molecular docking activities, should be conducted.

## Conclusion

Our study is the first to reveal the phytochemical activation of dopaminergic receptors and their regulatory roles in the browning of 3T3-L1 adipocytes and the ATP-dependent thermogenic process. This study may provide important evidence for comprehensive in vivo functional studies in the future. Overall, our findings show that the thermogenic action of ECH *via* the activation of DRD1 and DRD5 significantly contributed to beiging and ATP-consuming futile cycles that dissipate energy, which may be beneficial in the development of innovative approaches to obesity treatment.

## Supplemental Materials

Supplementary data for this paper are available on-line only at http://jmb.or.kr.

## Figures and Tables

**Table 1:** Primer sequences used for real-time quantitative RT-PCR.

Gene	Forward	Reverse
*Drd1*	AGGGGTTTTGGGAGAAGTGAC	AGTCACTTTTCGGGGATGCTG
*Drd5*	GGGGTTTTGGGAGAAGTGAC	AGTCACTTTTCGGGGATGCTG
*Cited1*	GGGGTAAAAGATCGCAAGGC	TGGTAGAAGGGGTGGCAGTA
*Ppargc1α*	ATGAATGCAGCGGTCTTAGC	AACAATGGCAGGGTTTGTTC
*Prdm16*	GATGGGAGATGCTGACGGAT	TGATCTGACACATGGCGAGG
*Tbx*1	AGCGAGGCGGAAGGGA	CCTGGTGACTGTGCTGAAGT
*Ucp1*	CCTGCCTCTCTCGGAAACAA	GTAGCGGGGTTTGATCCCAT
*Cd137*	GGTCTGTGCTTAAGACCGGG	TCTTAATAGCTGGTCCTCCCTC
*Cidea*	CGGGAATAGCCAGAGTCACC	TGTGCATCGGATGTCGTAGG
*Cebpa*	CCCTCTCCCCTAGTTGTCCA	CTTTCCAGACCCAGAGCCAG
*Pparγ*	ACCATGGTAATTTCAGTAAAGGGT	CCTGACGGGGTCTCGGT
*Mest*	AGCAGCTTTCCTCTGCGG	GATACAGGATCGGAGGTGGC
*Srbf1*	CCTGACAGGTGAAATCGGCG	GTTGTTGATGAGCTGGAGCA
*Fasn*	CACTGCCTTCGGTTCAGTCTC	ACACCCTCCAAGGAGTCTCAC
*Scd1*	TGGAGACGGGAGTCACAAGA	ACACCCCGATAGCAATATCCAG
*Slc2a4*	CTCTGACGTAAGGATGGGGA	ACCTTCTGTGGGGCATTGAT
*Lipe*	CTTGGGGAGCTCCAGTCGGAAG	TGTCTTCTGCGAGTGTCACCAG
*Pnpla2*	CTGCTGTAAACCCCTGGTCT	CCACGGATGGTCTTCACCAG
*Mfn1*	TCCCCTCTTTCGGGAGGATG	GTCCGGAGCTCGAAGGTCA
*Mfn2*	GACACGGGACGGTTACCAG	CTCTGAACGCTGTCACCTCA
*PDK4*	TGAACACTCCTTCGGTGCAG	TCGAACTTTGACCAGCGTGT
*Tfam*	TAGGCACCGTATTGCGTGAG	GTGCTTTTAGCACGCTCCAC
*Cpt1*	GACTCCGCTCGCTCATTCC	TTGAGGGCTTCATGGCTCAG
*Nrf1*	GCTAATGGCCTGGTCCAGAT	CTGCGCTGTCCGATATCCTG
*Cycs*	CACCGACACCGGTACATAGG	TAATTCGTTCCGGGCTGGTC
*Ryr2*	CAGAAGGGACTGCTCAAGGT	TCGAAGGCCAGTTTGTCAGT
*Tfam*	ATGTGGAGCGTGCTAAAAGC	GGATAGCTACCCATGCTGGAA
*Cox4*	ACATTCAGGGTGCCTCTTTG	CATGGCAGAAGTGGGAGATT
*Atp2a1b*	TCAGGGCAGGAGCATCATTC	ATGTAGCGCTGTCAGTCAAGA
