# Supplementary material for: Echinacoside Induces UCP1- and ATP-Dependent Thermogenesis in Beige Adipocytes via the Activation of Dopaminergic Receptors
Source: J Microbiol Biotechnol. 2023 Jul 17;33(10):1268–80. doi: 10.4014/jmb.2306.06041 (PMC10619551; doi:10.4014/jmb.2306.06041)
Supplement: Supplementary file 1 [file jmb-33-10-1268-supple.pdf]

J. Microbiol. Biotechnol.
https://doi.org/10.4014/jmb.2306.06041

Supplementary Table 1. List of antibodies used for western blot.

Primary antibody	Supplier	Catalog Number
ATGL	Santa Cruz Biotechnology	sc-365278
ACC	Santa Cruz Biotechnology	sc-30212
p-ACC	Santa Cruz Biotechnology	sc-271965
ATF2	Santa Cruz Biotechnology	sc-242
p-ATF2	Santa Cruz Biotechnology	sc-8398
AMPK	Santa Cruz Biotechnology	sc-398861
p-AMPK	Invitrogen	44-150g
ACOX1	Santa Cruz Biotechnology	sc-98499
C/EBPα	Santa Cruz Biotechnology	sc-365318
CPT1	Santa Cruz Biotechnology	sc-20670
CREB	Santa Cruz Biotechnology	sc-186
p-CREB	Santa Cruz Biotechnology	sc-81486
ERK1/2	Santa Cruz Biotechnology	sc-514302
pERK1/2	Santa Cruz Biotechnology	sc-7383
FAS	Santa Cruz Biotechnology	sc-55580
PKA	Santa Cruz Biotechnology	sc-98951
p38	Santa Cruz Biotechnology	sc-7149
p-p38	Santa Cruz Biotechnology	sc-7973
PPARγ	Santa Cruz Biotechnology	sc-7273
PGC-1α	Santa Cruz Biotechnology	sc-517380
UCP1	Santa Cruz Biotechnology	sc-293418
Actin	Invitrogen	MA5-11869

pHSL	Cell Signaling Technology	4137s
CYT-C	Abcam	ab110325
OXPHOS	Abcam	ab110413
VDAC	Santa Cruz Biotechnology	sc98708
MCU	Cell Signaling Technology	14997s
ATP5B	Santa Cruz Biotechnology	sc-55597
PPARa	Santa Cruz Biotechnology	sc-398394
SERCA2b	Cell Signaling Technology	D51B11
RyR2	Proteintech	19765-1-AP
CKmt	Proteintech	13207-1-AP
ɑ1-AR	Invitrogen	PA585077
PDE4	Abcam	ab14628
CamKII	Abcam	ab134041
**Secondary antibody**	**Supplier**	**Catalog Number**
mouse IgG	Santa Cruz Biotechnology	sc-2025
Rabbit IgG	Santa Cruz Biotechnology	sc-2027
